# Editorial: Deciphering immune responses in infectious and inflammatory pathology

**DOI:** 10.3389/fimmu.2026.1957450

**Published:** 2026-09-02

**Authors:** Nikunj Tandel

**Affiliations:** Department of Biotechnology, Veer Narmad South Gujarat University (VNSGU), Surat, Gujarat, India

**Keywords:** autoimmune disease, inflammation, memory, microenvironment, translational research

## Overview of contributions

The Research Topic “*Deciphering immune responses in infectious and inflammatory pathology*” examines how the immune system behaves during infection, inflammation, and autoimmune-like conditions, with support from clinical evidence. Collectively, these studies highlight the complex balance between protective immunity and damaging inflammation. Taken together, the findings suggest avenues for the design of next-generation, target-specific interventions.

## Host–pathogen interactions and infection-driven immunity

Several studies in this Research Topic focused on how pathogen exposure and chronic infection shape immune signaling and function. The contribution on the immune profiles of individuals living in areas endemic for infectious diseases (Coelho et al.) showed that repeated exposure to pathogens can alter immune responses and give rise to partially “educated” or memory-like immune states. These states could help the host in certain settings or may also hinder it. Endemic regions may see low-grade infections that could persist regardless of an active immune response.

A related study on sepsis-induced immunosuppression (Gao et al.) described how severe infection can push the immune system into a suppressed state through impaired antigen presentation and altered T-cell function, making the host vulnerable to secondary infections. These findings showed that the outcome of infection depends not only on the initial encounter with the pathogen and its clearance, but also on how immune responses are regulated post-encounter. These findings underscore the need to monitor immune dynamics beyond the acute phase of illness.

## Immune dysregulation and inflammatory pathology

Several studies in this Research Topic examined how pathogen exposure and chronic infection reprogram host immune responses. One study assessed immune profiles in individuals living in regions endemic for infectious diseases (Coelho et al.), showing that continuous or repeated exposure to pathogens can alter immune cells. This may act as a double-edged sword, influencing both susceptibility to infection and disease severity. These findings are consistent with broader observations from endemic settings, where immune phenotypes often reflect a balance between protection and low-grade chronic inflammation.

Another study focused on oral lichen planus (Cheng et al.), a chronic inflammatory mucosal disease whose pathogenesis remains poorly understood. Using single-cell RNA sequencing, the authors identified SFRP2^+^ fibroblasts as the source of inflammatory fibroblasts within the lesion. A subset of these cells expressed Wnt5a, which is associated with antigen processing and presentation pathways. In addition, SFRP2^+^Wnt5a^+^ fibroblasts appeared to sustain local inflammation through close interactions with CD8+ T cells and epithelial cells. This study also reported increased expression of pro-inflammatory and antigen presentation-associated molecules, including CXCL12, CXCL14, HLA-A, HLA-B, HLA-C, and ERAP2, in oral lichen planus samples. Taken together, these observations suggest that fibroblasts should not be viewed as purely structural cells in this disease. Rather, they appear to play an active role in shaping the inflammatory microenvironment and sustaining chronic immune activation.

## Infection-autoimmunity cross-talk and viral immunology

Several studies in this Research Topic explored the shared mechanisms underlying infectious and autoimmune diseases. One article, on the intersection of influenza infection and autoimmunity (Xie et al.), examined how viral infection may initiate or worsen autoimmune responses through mechanisms such as molecular mimicry, bystander activation, and tissue-specific inflammation, highlighting the idea that autoimmune phenomena often do not arise in isolation. Rather, they may result from multiple interacting factors, including infection-driven immune activation and underlying host susceptibility.

Another study examined the role of the Epstein-Barr virus (EBV) in the pathogenesis of multiple sclerosis (Ballerini et al.) using an EBV interactome-guided approach. The authors showed how persistent viral infection can influence immune cell signaling, trafficking, and resident immune responses in the central nervous system. Their findings support the current view that chronic viral infections may act as environmental triggers for neuroinflammatory diseases by shaping immune networks at local and systemic levels.

## Translational and clinical immunology

This Research Topic underscores the importance of immunological insights for diagnosis, prognosis, and therapeutic decision-making in clinical and translational research. The case report on IgG4-related kidney disease (Dong et al.) showed how integrating diverse clinical and immunological data can improve the management of complex immune-mediated diseases.

In addition, the article on thymosin-α1 discussed its role in modulating immune-inflammatory responses (Tian et al.) and suggested that selective immunomodulation could help control excessive inflammation and maintain protective immunity in critical care and post-infection settings. Together, these contributions emphasized that successful immunomodulation requires disease-specific strategies rather than broad immunosuppressive or stimulatory regimens. The integration of clinical phenotypes with immune-specific readouts, such as cytokine networks, immune cell subsets, and systemic immune profiles, may open new ways for developing precise and effective therapeutic interventions.

## Key insights and emerging trends

In summary, the most effective immunomodulation for infectious and inflammatory diseases depends on the disease context and the local microenvironment, where different immune sentinels play distinct roles. It is not enough to rely on broad immunosuppressive or stimulatory approaches. Rather, combining immunological readouts with multi-omics data and longitudinal studies would improve disease stratification and help define how the immune response changes over time. Similarly, machine-learning and artificial intelligence may help identify disease-specific immune signatures and support the development of personalized therapies.

## Conclusion

This Research Topic brings together studies that deepen our understanding of the patterns and functions of immune responses in infectious and inflammatory pathology. Together, the different clinical, systemic, and mechanistic approaches used in these articles highlight the complexity of immune regulation. This Research Topic also bridges the gap between basic immunology, translational research, and clinical practice. Insights from the experimental and field data presented here are expected to support the development of improved diagnostic tools, more targeted therapies, and refined preventive strategies. Ultimately, these contributions may help improve patient outcomes across a broad spectrum of infectious, autoimmune, and inflammatory diseases.

